# Fatty Acid β-Oxidation in Kidney Diseases: Perspectives on Pathophysiological Mechanisms and Therapeutic Opportunities

**DOI:** 10.3389/fphar.2022.805281

**Published:** 2022-04-20

**Authors:** Zhumei Gao, Xiangmei Chen

**Affiliations:** ^1^ Department of Nephrology, The Second Hospital of Jilin University, Jilin, China; ^2^ Department of Nephrology, The First Medical Center, Chinese PLA General Hospital, Beijing, China

**Keywords:** acute kidney injury, chronic kidney disease, diabetic nephropathy, fatty acid β-oxidation, mesenchymal stem cell therapy

## Abstract

The kidney is a highly metabolic organ and requires a large amount of ATP to maintain its filtration-reabsorption function, and mitochondrial fatty acid β-oxidation serves as the main source of energy to meet its functional needs. Reduced and inefficient fatty acid β-oxidation is thought to be a major mechanism contributing to kidney diseases, including acute kidney injury, chronic kidney disease and diabetic nephropathy. PPARα, AMPK, sirtuins, HIF-1, and TGF-β/SMAD3 activation have all been shown to play key roles in the regulation of fatty acid β-oxidation in kidney diseases, and restoration of fatty acid β-oxidation by modulation of these molecules can ameliorate the development of such diseases. Here, we disentangle the lipid metabolism regulation properties and potential mechanisms of mesenchymal stem cells and their extracellular vesicles, and emphasize the role of mesenchymal stem cells on lipid metabolism. This review aims to highlight the important role of fatty acid β-oxidation in the progression of kidney diseases, and to explore the fatty acid β-oxidation effects and therapeutic potential of mesenchymal stem cells for kidney diseases.

## 1 Introduction

Over the last 20 years, approximately 850 million people have been suffering from some form of kidney disease (Bharati and Jha, 2022). Many kidney diseases eventually progress to end-stage renal disease (ESRD) due to the lack of specific treatment drugs. To date, renal replacement therapy is the only effective treatment for ESRD, which imposes a very large economic burden on the health system and individuals. Therefore, there is an urgent need to find potential novel therapeutic targets and develop sustainable and effective strategies to prevent ESRD.

The kidney requires a tremendous amount of energy to maintain a stable internal environment of the body. Approximately 70% of the glomerular filtrate and its solutes are reabsorbed in the proximal tubules, so tubular epithelial cells (TECs) are mitochondrially enriched and primarily relying on fatty acid β-oxidation (FAO) as their energy source to meet their functional needs (‐). Fatty acids are converted into products, such as acetyl-CoA, to generate energy through fatty acid uptake, activation, transportation to the matrix, and FAO processes in the body. Dysregulation of lipid uptake and FAO mediated lipid deposition are thought to contribute to kidney diseases, including acute kidney injury (AKI), chronic kidney disease (CKD), and diabetic nephropathy (DN) (Li et al., 2020a; Rong et al., 2021; Li et al., 2022). An impaired FAO is thought to be a major mechanism underlying kidney injury.

Carnitine palmitoyl transferase 1A (CPT1A) is a rate-limiting enzyme in the FAO pathway. A recent study showed that CPT1A overexpression in kidney tubules significantly reduced renal fibrosis and protected against kidney function deterioration by restoring FAO function, preventing mitochondrial dysfunction, TEC differentiation, and increasing cellular oxidative capacity in 3 CKD animal models (Miguel et al., 2021a). This discoveries have expanded the understanding of the relationship between FAO and kidney injury.

Several reports have demonstrated that mesenchymal stem cells (MSCs) and their constituents, extracellular vesicles (EVs), are a novel therapeutic target for kidney diseases (Yea et al., 2021; Li et al., 2021a; Peng et al., 2022). Recently, it has been demonstrated that MSCs and MSC-EVs can regulate the lipid metabolism by increasing the expression of FAO-related genes and reducing fatty acid uptake (CD36) in metabolic diseases (Li et al., 2019a; El-Derany and AbdelHamid, 2021). However, the underlying mechanisms involving MSCs and FAO in kidney diseases remain poorly understood. Therefore, in this review, we focus on an in-depth discussion of the mechanisms and regulation of FAO in kidney diseases. In addition, we explore the effects of MSCs on FAO disorder, thereby aiding the application of MSCs in kidney diseases.

## 2 Fatty Acids Uptake in Kidney Diseases

Fatty acids (FAs) are the preferred substrate for proximal tubule ATP generation (Nieth and Schollmeyer.1966). In plasma, water-insoluble free FA are solubilized by complexing with albumin and transported to specific parts of the body for utilization. Although free FAs can enter the cytoplasm by passive diffusion, most of long-chain fatty acids (LCFAs) are taken up by transporters in proximal tubules. The cluster of differentiation-36 (CD36), FA-binding proteins (FABPs) and FA-transport proteins (FATPs) families are the most common and frequently studied FAs transporters in the kidney (Storch and McDermott.2009; Khan et al., 2018).

### 2.1 Cluster of Differentiation-36

CD36, also known as fatty acid translocase, is a scavenger receptor capable of binding many ligands, and is responsible for cellular uptake of long-chain fatty acids (Coburn et al., 2000). Human CD36 overexpression in the proximal tubules is associated with aggravation or progression of AKI after folic acid treatment (Jung et al., 2018). An increased expression of CD36 and CD36-mediated lipid deposition was also observed in the kidney of CKD and DN (Okamura et al., 2007).

CD36 is an important receptor for macrophages to recognize and phagocytize apoptotic cells and oxidized lipids. The severity of renal fibrosis in macrophage CD36-deficient mice was significantly lower than that in wild mice. This difference was associated with the attenuation of oxidative and proinflammatory pathways that promote fibrogenesis in CKD (Okamura et al., 2009; Pennathur et al., 2015).

Renal CD36 is also highly expressed in TECs (Susztak et al., 2005). An high glucose stimulated CD36-dependent Wnt/β-catenin activation in TECs is related to advanced oxidation protein product-induced lipid accumulation, which is thought to contribute to renal fibrosis (Li et al., 2019b). In addition, an elevated CD36 expression in TECs promotes mtROS production, apoptosis, epithelial-mesenchymal transition, and inflammatory responses, which in turn promote DN progression (Susztak et al., 2005; Hou et al., 2015; Hou et al., 2021). Interestingly, Susztak et al. generated a mouse model with kidney-specific overexpression of CD36 and found that FA accumulation was already evident in 8-week-old CD36-transgenic animals, while markers of fibrosis increased by 20 weeks of age (Kang et al., 2015). Thus, CD36 plays an important roles in kidney diseases, but an increased expression of CD36-induced lipid accumulation is not a major contributor to renal fibrosis. Moreover, the ubiquitous expression and cell-specific effects of CD36 must be considered when development of novel peptides that target CD36.

### 2.2 FA-Binding Proteins and FA-Transport Proteins 2

FABPs bind long-chain FAs with high affinity and play a central role in coordinating lipid transport and metabolism (Storch and McDermott, 2009), which comprises at least nine homologous proteins with similar tertiary structures and specific tissue distribution patterns (Li et al., 2020b). Liver-FABP (L-FABP) is expressed in the proximal tubules of both the normal and diseased human kidney (Maatman et al., 1991; Kamijo-Ikemori et al., 2011), which facilitates the excretion of lipid peroxidation products from TECs, promotes FAO and then attenuates tubulointerstitial damage to achieve renoprotection (Xu et al., 2015). Numerous studies have demonstrated that L-FABP is a promising biomarker for several kidney diseases, and it has also been shown that upregulation of renal L-FABP can protect renal function in human L-FABP transgenic mice after cisplatin treatment (Negishi et al., 2008; Manabe et al., 2012).

Adipose-FABP (also known as FABP4) has been reported to be significantly elevated in many AKI models, and inhibition of FABP4 can attenuate endoplasmic reticulum stress, mitochondrial dysfunction, inflammation, and apoptosis and restore kidney function (Huang et al., 2018; Tan et al., 2019; Li et al., 2021b). Inhibition of FABP4 can also enhance FAO and rebalance abnormal lipid metabolism in TECs and attenuate the progression of kidney fibrosis (Chen et al., 2021).

FATP2, which is encoded by *Slc27a2* and is localized most prominently on the proximal tubule apical membrane, is another protein that is implicated in the uptake of lipids in the kidney (Khan et al., 2018). It has been proven that FATP2 promotes renal interstitial fibrosis by inhibiting FAO in TECs, and inhibition of FATP2 could improve kidney function and alleviate fibrotic responses in a UUO kidney and DN (Khan et al., 2020; Chen et al., 2020).

In summary, the transport of FAs in the kidney exhibits unique characteristics in specific cells. In particular, FABP members exhibit unique functions in different tissues and microenvironments. Further understanding as to how the transport of FAs are specifically regulated in different cells will provide novel insights into the transport actions of FAs and facilitate their applications in kidney diseases.

## 3 Fatty Acids β-Oxidation in Kidney Diseases

FAs are activated to acyl-CoA before entering the mitochondria for complete β-oxidation. CPT1 is present in the outer membrane of mitochondria, catalyzes the synthesis of acyl carnitine from long chain fatty acyl CoA and carnitine. Then, under the action of carnitine-acylcarnitine translocase, the acyl carnitine enters the mitochondrial matrix and is transformed into acyl-CoA under the action of carnitine acyl transferase II in the inner membrane of mitochondria.

FA β-oxidation is the core process of FA catabolism and depends on the concerted action of both peroxisomes and mitochondria (Fransen et al., 2017). Peroxisomes and mitochondria contain different enzymes for β-oxidation, which preferentially oxidize short- and medium-chain FAs, whereas peroxisomes metabolize very long-chain FAs (Vasko.2016). These very-long-chain FAs are transported to the peroxisome matrix through ATP-binding protein ABCD1-3, which shortens the FA chain after several rounds of peroxisomal β-oxidation, and are then transferred into mitochondria for complete FAO (Lodhi and Semenkovich.2014; He et al., 2021). Acyl-CoA undergoes a β-oxidation reaction to form acetyl-CoA, which is completely oxidized by the tricarboxylic acid cycle.

### 3.1 Regulation of Fatty Acid β-Oxidation

#### 3.1.1 Peroxisome Proliferators-Activated Receptor α

Peroxisome proliferators-activated receptor α (PPARα) activation induces FAO and is most prominently expressed in the adult kidney (Braissant et al., 1996; Montaigne et al., 2021). The assembled complex containing PPRE/PPARα/RXRα/ligands/coactivators controls the expression of the genes involved in FAO (Libby et al., 2021).

Regulation of PPARα activity factors, including PPARα gene expression and protein translation, ligand specificity and availability, cofactor recruitment, corepressors or coactivators, and posttranslational modification, is an effective strategy to prevent and treat lipid disorders in kidney diseases (Bougarne et al., 2018). Blanquart demonstrated that synthetic PPARα ligands such as Wy14643, GW7647, or fibrates can increase the stability of PPARα, thereby regulating the transcription of PPARα target genes (Blanquart et al., 2002). In addition, PPARα ligands fibrates can also directly activate PPARα to induce the expression of fatty acid metabolism-related genes (Staels et al., 1998). Peroxisome proliferator-activated receptor γ coactivator 1α (PGC-1α) is a important coactivator of PPARa, and regulates FAO by increasing the activity of PPARα in the proximal tubule epithelial cells (Vega et al., 2000; Portilla et al., 2002).

#### 3.1.2 Adenosine Monophosphate-Activated Protein Kinase

Adenosine monophosphate-activated protein kinase (AMPK) is a master regulator of metabolism, which restores energy balance during metabolic stress both at the cellular and physiological levels (Garcia and Shaw.2017). Liver kinase B1 (LKB1) is the upstream kinase of AMPK, causing an increased Thr172 phosphorylation and AMPK activation under energy stress conditions (Lin and HardiE, 2018). Malonyl-CoA is a potent inhibitor of CPT1, activated LKB1/AMPK inhibits the conversion of acetyl-CoA to malonyl-CoA by phosphorylating acetyl-CoA carboxylase 1 (ACC1) and ACC2, which in turn promotes FAO (Jeon et al., 2012). Moreover, AMPK initiates many important gene regulatory functions in skeletal muscles by directly phosphorylating PGC-1α protein at threonine-177 and serine-538 (Jager et al., 2007).

#### 3.1.3 TGF-β/SMAD3

TGF-β is an important upstream modulator of fatty acid metabolism (Kang et al., 2015). TGF-β-driven SMAD3-dependent manner inhibit the expression of PGC-1α and downstream lipid deposition (Yadav et al., 2011). Moreover, TGF-β/SMAD3 signaling pathways reduce PPARα target gene expression and lower the rates of FAO in cultured myocytes by suppressing PPARα activity (Sekiguchi et al., 2007).

#### 3.1.4 Sirtuins

Sirtuins are a family of NAD^+^-dependent deacylases that exhibit a broad range of cellular functions. SIRT3 is localized in the mitochondrial matrix, where it promotes fatty acid oxidation in the liver and heart by deacetylating enzymes, such as long-chain acyl-CoA dehydrogenase (LCAD) (Hirschey et al., 2010). Unlike SIRT3, SIRT5 is also present in peroxisomes. It was observed that SIRT5 promotes the efficiency of mitochondrial FAO by increasing the activity of enoyl-CoA hydratase (ECHA) in the heart (Sadhukhan et al., 2016). However, in contrast to mitochondria, SIRT5 suppresses peroxisomal FAO by inhibiting ACOX1 activity *in vitro* and in rodent liver (Chen et al., 2018).

#### 3.1.5 Hypoxia-Inducible Factor 1

Hypoxia-inducible factor 1(HIF1) is a key transcription factor regulating lipid metabolism and mediating inhibition of PPARα expression during hypoxia (Narravula and Colgan.2001). CPT1A is a direct target gene of HIF and is repressed by HIF1 and HIF2, thereby reducing fatty acid transport into the mitochondria, and forcing fatty acids to lipid droplets for storage (Du et al., 2017).

### 3.2 Defective Fatty Acid β-Oxidation in Kidney Diseases

#### 3.2.1 Acute Kidney Injury

Reduction of FAO-related metabolic enzymes (CPT1 and medium chain–specific acyl-CoA dehydrogenase) and increased intrarenal lipid accumulation in proximal tubule cells were observed during ischemia reperfusion- and cisplatin-induced AKI (Portilla et al., 2000; Portilla et al., 2002; Tran et al., 2016). It is suggested that nucleocytoplasmic shuttling of PPARα is involved in lipid disorders in AKI. PPARα is a dynamic shuttling between the cytosol and the nucleus under physiological conditions and active PPARα is mainly located in the nucleus (Bougarne et al., 2018). The mitochondrial matrix protein cyclophilin D in mitochondria binds to PPARα and sequestration lead to inhibition of its nuclear translocation and transcription of PPARα-regulated FAO genes during cisplatin-induced AKI (Jang et al., 2020). Sirtuins also play an important role in FAO regulation in AKI. In addition to regulating FAO by modulating PPARα expression, SIRT3 further regulates FAO by deacetylating the LBK1 and activating the AMPK signaling pathway in cisplatin-induced AKI (Li et al., 2020a). In contrast to SIRT3, loss-of-function of SIRT5 was renoprotective in AKI. SIRT5 regulates the balance between mitochondrial and peroxisomal FAO in proximal tubular epithelial cells, and SIRT5 deficiency appears to be protective by increasing peroxisomal FAO to protect against injury in ischemia-induced or cisplatin-induced AKI (Chiba et al., 2019).

#### 3.2.2 Chronic Kidney Disease

Genome-wide unbiased transcriptomic analysis revealed that FAO-related key metabolic enzymes and FAO transcriptional regulator factors were markedly decreased in CKD (Kang et al., 2015). In ischemia reperfusion-induced CKD, activated transcription factor of the unfolded protein response ATF6α inhibited PPARα expression, resulting in the downregulation of FA β-oxidation and mitochondrial dysfunction, and consequent lipid deposition and apoptosis induction, which eventually accelerated profibrogenic phenotypes (Jao et al., 2019). TGF-β1-induced and SMAD3-mediated repression of PGC1-α also play a critical role in the regulation of FAO in TECs (Kang et al., 2015). Moreover, LKB1 and AMPK are further important controllers of FAO in TECs. It was observed that deletion of LKB1 in renal tubular epithelial cells downregulated the transcript levels of rate-limiting enzymes in the β-oxidation pathway via the AMPK signaling pathway, and an impaired LKB1 led to CKD (Han et al., 2016). HIF-1α directly regulates the expression of PPARα and CPT1α. In CKD, an upregulated mitochondrial uncoupling protein 2 (UCP2) protein increased HIF-1α stabilization, which in turn stimulated lipid deposition and extracellular matrix accumulation and promoted fibrosis (Ke et al., 2020).

Several microRNAs have been shown to be involved in the pathophysiology of renal fibrosis. Recently, a study employing an microRNA array-based strategy indicated that both miR-150 and miR-495 are upregulated in UUO-induced fibrotic kidneys, and that microRNAs are capable of reducing the expression of CPT1A, PGC1-α, and TFAM mitochondrial function-related genes, leading to renal epithelial cell dedifferentiation and a TGF-β1-induced fibrosis phenotype (Miguel et al., 2021b). Notably, miR-21, which has been extensively studied in renal fibrosis, is highly expressed in kidney fibrosis (Chau et al., 2012). Indeed, miR-21 efficiently decreases PPARα expression, impairs FAO, and aggravates renal fibrosis development during aging (Chau et al., 2012; Chung et al., 2018).

#### 3.2.3 Diabetic Nephropathy

Studies have also revealed that mouse models with DN display a lower expression of key enzymes and regulators of FAO, and a higher intracellular lipid deposition than controls (Lee et al., 2020). Advanced glycation end products (AGEs) are thought to be involved in this pathogenesis, which induced a decrease in CPT2 expression that led to mitochondrial FAO damage and, eventually, renal fibrosis and DN (Lee et al., 2020).

### 3.3 Targeting Fatty Acid Oxidation in Kidney Diseases

Defective FAO is closely linked to the pathogenesis and progression of kidney diseases. Hence, relieving lipid accumulation could be an effective strategy to suppress the progression of kidney diseases.

Some studies have shown that restoration of PPARα activity and/or expression is a potential treatment strategy for preventing the progression of kidney diseases. PPARα agonists are the most treatable option targeting defective FAO. In cisplatin-induced AKI, PPARα ligand Wy-14643 (WY) prevented cisplatin-induced reduction of mRNA levels and the enzyme activity of mitochondrial medium chain acyl-CoA dehydrogenase (MCAD), and rescued MCAD-mediated FAO to ameliorated acute tubular necrosis (Li et al., 2004). PPARα agonists also had a very excellent effect in the CKD model. For example, treatment with fenofibrate restored FAO-related enzyme expression, improved renal function, and reduced kidney injury and fibrosis in a folic acid- and UUO-induced kidney fibrosis model (Kang et al., 2015). Similar results were observed after treatment with BAY PP1, a new PPARα agonist, in UUO and 5/6 nephrectomy models of renal fibrosis (Boor et al., 2011). Moreover, PPARα/β activator MHY2013 can significantly increase the expression of FAO-associated genes and prevent renal fibrosis during aging (Chung et al., 2020).

Activation of AMPK is another effective way to restore FAO. LKB1-deficient cells treated with an AMPK agonist (A769662) restored the levels of CPT1, CPT2, and ACOX1 in FAO and reduced apoptosis and dedifferentiation (Han et al., 2016). Moreover, it has been reported that metformin could reduce renal fibrosis by activation of energy-sensing kinase AMPK, thereby increasing phosphorylation of ACC and altering fatty acid metabolism (Lee et al., 2018). Uncoupling protein (UCP-1) located in the inner mitochondrial membrane inhibits the progression of acute renal injury by promoting the AMPK/ULK1/autophagy pathway (Xiong et al., 2021).

Indeed, the delivery of microRNA mimics or inhibitors has been proposed as a promising therapeutic strategy to prevent the development of renal fibrosis (Petrillo et al., 2017). For example, treatment with miR-9-5p was shown to reverse the downregulation of the expression of PPARα and PGC1A and FAO-related enzymes in a mouse model of UUO, further protecting against renal fibrosis (Fierro-Fernandez et al., 2020). In addition, miR-21 inhibition was shown to prevent CKD, which may be related to the enhancement of PPARα/RXR activity and improved mitochondrial function (Gomez et al., 2015). The details of intervention in fatty acid β-oxidation in AKI and CKD are summarized in Tables 1, 2.

**TABLE 1:** Intervene fatty acid β-oxidation in AKI.

Model	Treatment	Regulatory factor	Effect on kidney injury	Reference
Cisplatin	Genetic or pharmacological inhibition of cyclophilin D	PPARα activity↑Fatty acid oxidation↑Lipid accumulation↓	Mitochondrial function↑ Apoptotic↓ Inflammation↓ Cell cycle G2/M arrest↓	Jang et al. (2020)
Cisplatin	SIRT3 agonist	LKB1-AMPK pathway↑ Fatty acid oxidation↑ Lipid accumulation↓	Mitochondrial function↑ Renal function↑ Necrosis↓	Li et al. (2020b)
Cisplatin/Ischemic AKI	SIRT5-deficient mice	Peroxisomal fatty acid oxidation↑	Tubular injury↓ Oxidative stress↓ Renal function↑	Chiba et al. (2019)
Cisplatin	UCP1 agonist	AMPK/ULK1 pathway↑Lipid accumulation↓	Apoptotic↓ Inflammation↓ Autophagy↑	Xiong et al. (2021)
Cisplatin	PPARα ligand	PPARα activity↑ Fatty acid oxidation↑ Pyruvate dehydrogenase kinase-4 (PDK4)↑	Apoptotic↓ Inflammation↓ Necrosis	Li et al. (2004)

**TABLE 2:** Intervene fatty acid β-oxidation in CKD.

Model	Treatment	Regulatory factor	Effect on kidney injury	Reference
Folic acid	Overexpression of PGC1-α; Fenofibrate (PPARα agonist); Etoxomir (inhibitor CPT)	PGC1-α, CPT, PPARα Fatty acid oxidation↑	Fibrosis↓ Apoptosis↓ Renal function↑ Tabular injury↓	Kang et al. (2015)
UUO; 5/6 nephreetomized rats	BAY PPI (PPAR-α against)	PPAR-α↑ TGF-β1 expression↓	Fibrosis↓ Renal function↑ Interstital cell proliferation↓	Boor et al. (2011)
Tubule epithclial Lkb1 deletion mice	Fenofibrate A769662 (A MPK against)	PPARα↑ AMPK↑	Fibrosis↓ Apoptosis↓ Dedifferentiation↓	Han et al. (2016)
Unilateral ischemia-reperfusion injury	Atf6α-/- mice fenofibrate	PPARα↑ Lipid accumulation↓	Apoptosis↓ Fibrosis↓	Jao et al. (2019)
UUO	Mir-9-5p	PGC-1α↑ Fatty acid metabolism↓	Fibrosis↓ Mitochondrial function↑ apoptosis↓ Inflammation↓	Fierro-fermandez et al. (2020)
Alport nephropathy	Anti-microRNA-21	PPARα/RXR activity↑ PGC-1α↑ Fatty acid metabolism	Fibrosis↓ Mitochondrial function↑ Oxidative stress↓ Inflammation↓	Gomez et al. (2015)
Ischemia-representation injury; Folic acid nephropathy (FAN); Aristolochic acid nephropathy (AAN)	UCP2-deficient mice; Genetic inhibition of HIF-1α	PPARα↑ CPT1A↑ Lipid accumulation↓	ECM accumulation↓ Mitochondrial function↑	Ke et al. (2020)
UUO; FAN; Adenine-induced nephrotoxicity	CPC1A-knockin mice	Fatty acid metabolism↑	Fibrosis Mitochondrial function↑ Apoptosis↓ Inflammation↓ Cell cycle arrest↓	Miguel et al. (2021a)

## 4 Fatty Acid β-Oxidation and Stem Cell-Based Therapy

MSCs are pluripotent stem cells with the ability of self-renewal and multidirectional differentiation that have been isolated from various biological tissues (Naji et al., 2019; Zhou et al., 2021).

The regulation of lipid metabolism by MSCs and/or their EVs is well explored in cancer and metabolic diseases. Increasing evidence suggests that MSCs improve the stemness and drug-resistance of cancer cells through regulating CPT1 expression and FAO, which is mediated by the miR-3619-5p/AMPK/PGC1α/CEBPB axis in gastric cancer (Wu et al., 2020; Han et al., 2021). In a non-alcoholic steatohepatitis animal model, treatment with bone marrow-derived MSCs and their EVs significantly improved liver steatosis and ballooning in rats (El-Derany and AbdelHamid. 2021). Further mechanistic studies have shown that MSC-EVs could reduce the expression of fatty acid uptake and the synthesis genes CD36, SREB1, SREB2 and ACC, upregulate the gene expression levels of PPARα and the FAO metabolic enzyme CPT1, and increase mitophagy genes (Parkin, PINK1, ULK1, BNIP3L, ATG5, ATG7, ATG12) to improve nonalcoholic steatohepatitis (El-Derany and AbdelHamid. 2021). Moreover, MSCs and EVs can modulate lipid metabolism by activating the AMPK signaling pathway. For example, in diet-induced obesity mice, AD-MSCs treatment inhibited ACC1 activity by increasing the expression and phosphorylation of AMPK, which in turn promoted FAO and regulated lipid metabolism disorders (Liu et al., 2016). Furthermore, human amniotic MSC-EVs significantly inhibited high-fat diet-induced obesity by inhibiting lipid synthesis, activating AMPK and increasing UCP1/PPARα/PGC1α-regulated lipid consumption (Tan et al., 2021).

It is worth noting that different tissue-derived MSCs can confer different therapeutic efficacy in ameliorating lipid metabolic disorders in diabetic animals (Ma et al., 2021). Umbilical cord Wharton’s jelly (UC-MSCs) showed the strongest efficacy in reducing serum low-density lipoprotein cholesterol (LDL-C) levels, but AD-MSCs showed an very weak effect on LDL-C and substantially reduced lipid deposition in the liver (Ma et al., 2021). Moreover, lipid metabolic disturbance in diet-induced obesity mice was significantly alleviated after AD-MSC treatment but not in the UC-MSC treated group (Liu et al., 2016).

Unfortunately, few experimental studies have identified the impact of MSCs on lipid metabolism in kidney diseases. In a rat renal ischemia reperfusion model (right nephrectomy was performed and left renal ischemia lasted for 45 min) with, MSCs injection 7 days before surgery, after 48 h reperfusion, it was observed that MSCs administration induced the activation of the PPARα pathway and decreased the availability of free FAs, which in turn prevented lipid peroxidation and attenuated renal I/R damage (Erpicum et al., 2017). Given the importance of mitochondrial function in FAO and kidney diseases (Forbes and Thorburn, 2018; Chung et al., 2019; Jiang et al., 2020), we summarize the function and mechanism of MSCs in mitochondrial homeostasis in kidney diseases below.

MSCs play a protective role in mitochondria for renal repair mainly through the following mechanisms: 1) transfer of mitochondria to damaged proximal tubular epithelial cells (Konari et al., 2019); 2) regulation of mitochondrial biogenesis by enhancing PGC1-α expression, NAD + biosynthesis, and SIRT3 activity (Perico et al., 2017); 3) inhibition of mitochondria-mediated apoptosis and mitophagy (in hexavalent chromium-injured kidney) (Yin et al., 2019); and 4) activation of mitophagy (in sepsis- and cisplatin-induced AKI) (Wang et al., 2017; Guo et al., 2021). Recent studies have indicated that MSC-EVs also have a protective effect on mitochondrial damage caused by AKI, which protects TECs against injury by reducing mitochondrial fragmentation, normalizing mitochondrial membrane potential, and reversing mitochondrial DNA deletion and oxidative phosphorylation defects (Cao et al., 2020; Zhao et al., 2021).

In conclusion, MSCs and MSC-EVs can promote FAO and regulate lipid metabolism disorders, and can also effectively improve mitochondrial dysfunction in kidney diseases. However, relatively few studies have identified the impact of MSCs on lipid metabolism in kidney diseases. Future research in this area will help deepen our understanding of the metabolic regulation mechanism of MSCs therapy for kidney diseases and help discover more potential therapeutic targets.

## 5 Summary and Future Perspectives

**FIGURE 1:**
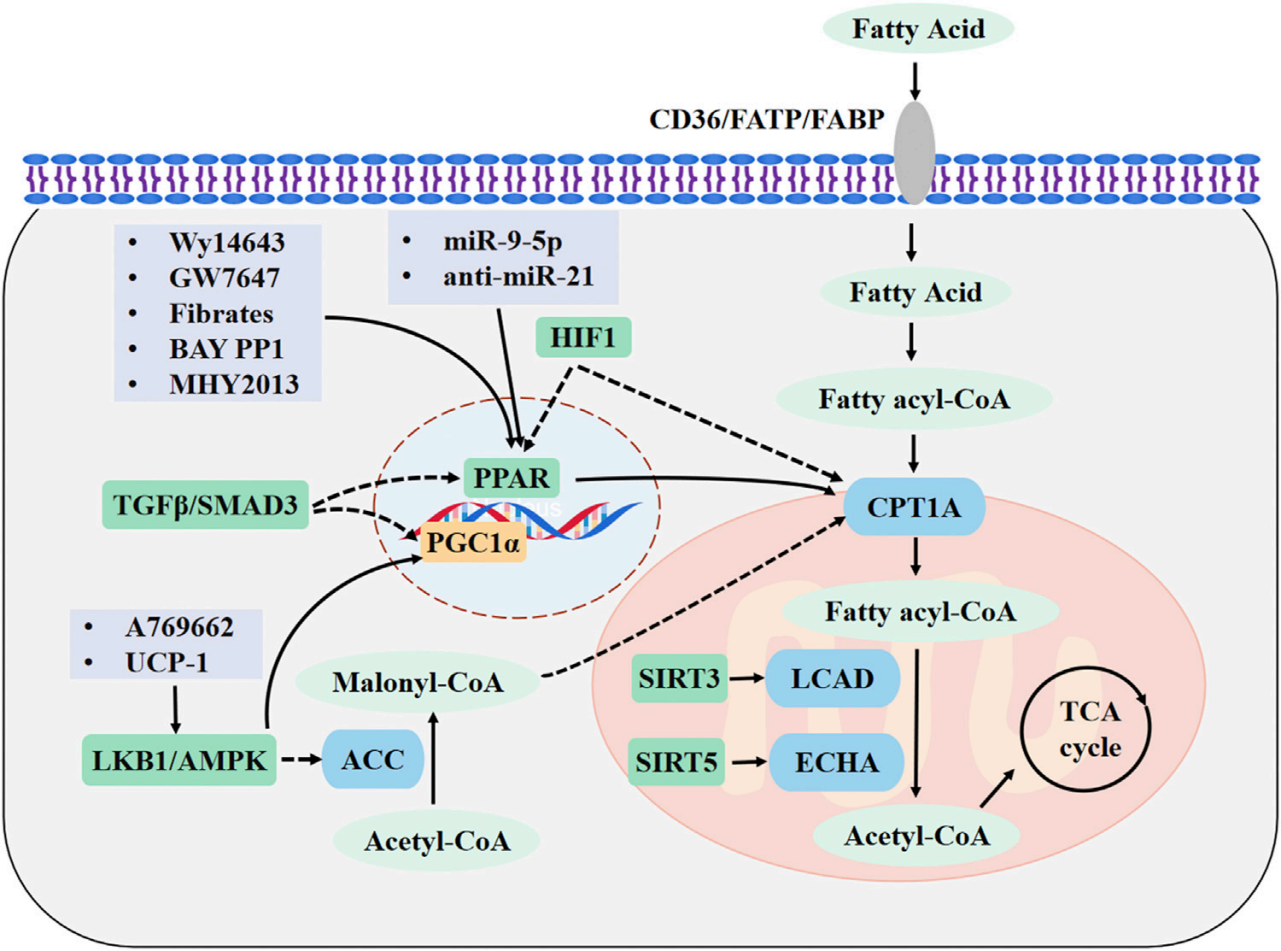
Major FAO pathways involved in kidney diseases and drug targets. Fatty acids (FAs) enter the cytoplasm through CD36, FABPs, or FATPs. In the cytosol, FAs are activated to fatty acyl-CoA and transported into the mitochondrial matrix through CPT1A. The acyl-CoA undergoes a β-oxidation reaction to form acetyl-CoA, which is completely oxidized by the tricarboxylic acid cycle. FAO is promoted by upregulating CPT1A and PPAR signaling. Drugs are highlighted in light purple boxes. Enzymes are shown in blue and metabolites are shown in light green. Regulatory molecules are shown in green boxes. FAO, fatty acid β-oxidation; CPT1A, carnitine palmitoyl transferase 1A; FABP, FA-binding protein; FATPs, FA-transport protein; PPAR, peroxisome proliferators-activated receptor; PGC-1α, peroxisome proliferator-activated receptor γ coactivator 1α; AMPK, adenosine monophosphate-activated protein kinase; LKB1, Liver kinase B1; ACC, acetyl-CoA carboxylase; LCAD, long-chain acyl-CoA dehydrogenase; ECHA, enoyl-CoA hydratase; SIRT3, sirtuin 3; SIRT5, sirtuin 5.

In this review, we have summarized the most recent findings on the pathophysiological mechanisms of FAO in kidney diseases. The evidences demonstrates that FAO is damaged in AKI, CKD and DN, and that recovery of FAO can effectively protect against kidney dysfunction. The HIF-1, TGF-β/SMAD3, and LBK1/AMPK signaling pathways can directly or indirectly affect the expression of key FAO metabolic enzymes. In addition, PPARα, sirtuins, and microRNAs are involved in the regulation of FAO. Thus, direct regulation of the key metabolic enzymes of FAO, such as CPT1A, or increasing the activity of their regulatory factors can significantly restore FAO and promote the recovery of kidney function. The major FAO pathways involved in kidney diseases and drug targets are shown in Figure 1.

One of the most important issues in kidney diseases is that each nephron segment has distinct physiological characteristics, consistent with different enzyme activities, protein abundances, and substrate utilizations (Tian and Liang, 2021). Moreover, in the context of kidney diseases, disruption of the local microenvironment promptly initiates changes in cellular metabolism and phenotypic changes (Basso et al., 2021; Dumas et al., 2021). Therefore, it is very important to elaborate the dynamic changes in the cellular metabolism of different nephron segments in the process of disease development. Furthermore, AKI can have different etiologies that are conditioned by different pathophysiological mechanisms (Ronco et al., 2019). Different disease models may have different metabolic characteristics. It has been reported that metabolic alterations precede changes in serum creatinine in cisplatin-induced nephrotoxicity (Portilla et al., 2006), but whether changes in metabolites can represent a potential marker for early diagnosis and progression of kidney diseases needs to be explored further.

Moreover, emerging experimental evidence suggests that the protective effect of MSCs and MSC-EVs on kidney diseases is partly attributed to their ability to regulate energy metabolism. However, few studies have explored the relationship between MSCs and FA metabolism. Hence, additional experimental studies are warranted to deepen our understanding of the energy metabolism mechanisms of MSC therapy and to discover additional potential therapeutic targets for AKI and CKD**.**

## Abbreviations

ACC, acetyl-CoA carboxylase; AKI, acute kidney injury; AMPK, adenosine monophosphate-activated protein kinase; CKD, chronic kidney disease; CPT1A, Carnitine palmitoyl transferase 1A; DN, diabetic nephropathy; ECHA, enoyl-CoA hydratase; ESRD, end-stage renal disease; EVs, extracellular vesicles; FAs, fatty acids; FABPs, FA-binding proteins; FAO, fatty acid β-oxidation; FATPs, FA-transport proteins; LCAD, long-chain acyl-CoA dehydrogenase; L-FABP, Liver-FABP; LKB1, Liver kinase B1; MCAD, medium chain acyl-CoA dehydrogenase; MSCs, mesenchymal stem cells; PGC-1α, peroxisome proliferator-activated receptor γ coactivator 1α; PPARα, peroxisome proliferators-activated receptor α; SIRT3, sirtuin 3; SIRT5, sirtuin 5; TEC, tubular epithelial cell; UCP, uncoupling protein.
